# Human papillomavirus vaccination in view of the National Cancer Control Plan 2020-2029 in Morocco

**DOI:** 10.11604/pamj.2023.45.115.40004

**Published:** 2023-07-04

**Authors:** Mohamed Zraidi, Mohammed Ibriz

**Affiliations:** 1 Reference Center for Reproductive Health, Regional Hospital Center, Kenitra, Morocco,; 2 Plant, Animal and Agro-industry Production Laboratory, Biology Department, Faculty of Sciences, Ibn Tofail University, Kenitra, Morocco

**Keywords:** HPV, vaccination, cervical cancer, Morocco

## Abstract

In Morocco, cervical cancer represents a major public health problem. It ranks second, after breast cancer in Moroccan women, in terms of incidence and mortality. Each year, more than 3,300 new cases and nearly 2,500 deaths are recorded. A Moroccan national program to fight against cervical cancer (CC) based on the practice of visual inspection after application of acetic acid was set up in 2010, allowing screening and possibly of immediate treatment of premalignant lesions of the cervix. In September 2022, a partnership circular between the Ministry of Health and Social Protection (MSPS) and the Ministry of National Education, Preschool and Sports was launched. it announces that the MSPS will integrate the HPV vaccine using the quadrivalent vaccine into the Moroccan National Immune Program (PNI), the vaccination will be aimed to 11-year-old girls, mainly those in school, who will receive two doses separated by at least a period of 6 months.

## Brief

The cervical cancer screening program in Morocco has been institutionalised since 2012. The screening test uses the VIA test for women who is 30-49 years old. It is intended in the National Cancer Prevention and Control Plan framework 2020-2029 to switch to HPV testing for women who is 30 and 40 years old, according to the application of the WHO recommendations which advocate the need to switch to a more efficient screening test [1]; it is why a technical feasibility study of HPV screening has been launched in three Moroccan regions. By the way, the HPV vaccination has been launched in October 2022 [2].

The vaccination using the quadrivalent vaccine has joined the arsenal to eradicate cervical cancer given its effectiveness. Indeed, a literature review of studies measuring the burden of HPV-associated disease and infection in Australia before and after the introduction of HPV vaccine showed that vaccination with the quadrivalent vaccine had a significant impact on HPV-related disease. Of nearly 1,544 HPV-associated cancers in 2012, 1,242 were prevented by the quadrivalent vaccine and a further 187 ano genital cancers could have been prevented by the nanovalent vaccine [3]. Globally, 50-80% of sexually active women acquire an HPV infection at least once in their lifetime [4,5]. Fortunately, almost HPV infections disappear, resolving through the natural immune response and becoming undetectable after 6-18 months [6,7]. However, if the infection persists, it can cause pre-cancerous cells that can, in the long term, become cancerous.

In 2020, the recent estimate of cervical cancer incidence and mortality according to the Global Cancer Observatory indicate that there are approximately 2,165 new cases (an incidence rate of 10.4 per 100,000 women) [8]. According to the same research, the cervical cancer annual deaths rate is approximately 1,199 women in 2020, or 5.8 new deaths per 100,000 [9]. According to the International Agency for Research on Cancer (IARC), cervical cancer is the second most common cancer and the second leading cause of death. It ranks third among young Moroccan women who are 15-44 years old following breast and thyroid cancer, with a crude incidence rate of 7.5 per 100,000 women. Furthermore, according to the IARC/ICO 2019 report, an estimated 13.2 million women in Morocco who is 15 years or older are at risk to be affected by cervical cancer, therefore, the incidence of cervical cancer in Morocco and the death rate concerning this cancer are the highest in North Africa [9]. Thus, if the rate remains as it is nowadays, the forecast for the years 2030 and 2040 foresees a raise to nearly 2835 new cases of cervical cancer by 2030, that is 30.9% more than the rates in 2020, as it happens a rate of 13.8 per 100,000 women.

The vaccine against HPV infections has been introduced in many countries around the world since 2006/2007, although coverage until now is variable, depending on geographical area and human development index. In 2020, WHO reports that less than a quarter of low-income countries have introduced the HPV vaccine into their national immunisation schedule, while more than 85% of high-income countries have done so [10]. The percentage of countries with routine HPV vaccination programmes at the end of June 2020 was 31% in Africa, 40% in Asia, 56% in Oceania, 77% in Europe and 85% in the Americas [10]. HPV vaccination, given before exposure to the viruses, can prevent infection with the targeted types. For this reason, vaccination is recommended for teenagers who are not yet sexually active.

The Kingdom of Morocco has quite respectful experience in eliminating diseases that cause serious public health problems, such as malaria, blinding trachoma, schistosomiasis, leprosy, and certain vaccine-targeted diseases such as diphtheria, neonatal tetanus, and poliomyelitis. It has therefore the ability to meet the challenge of eliminating cervical cancer. Indeed, in September 2022 a directive circular signed by the Health and Social Protection Ministry and the National Education, Pre-school and Sports Ministry, was launched announcing that an HPV vaccine will be integrated in the Moroccan National Immunisation Programme (PNI), using the quadrivalent one. The vaccination will be aimed at schooled girls aged 11 years, mainly those in school, who will receive 2 doses separated by at least 6 months [2].

To conclude, the fight against cervical cancer in Morocco, initially based on visual inspection with acetic acid while waiting to switch to screening by HPV genomic test as suggested by the WHO. The treatment of cervical precancerous lesions basically by the thermo coagulation and alternately by loop electro excision procedure (LEEP), or even by surgery conisation with scalpel blade, is currently completed by the anti HPV vaccination, using the quadrivalent vaccine. In addition, countries with more than 70% vaccination coverage are those that have organised screening. Conversely, countries that have organised neither screening, nor vaccination have a vaccination coverage rates below 50%.
